# Empathy, social media, and directed altruistic living organ donation

**DOI:** 10.1111/bioe.12438

**Published:** 2018-03-15

**Authors:** Greg Moorlock, Heather Draper

**Keywords:** altruism, empathy, kidney, organ donation, social media, transplant

## Abstract

In this article we explore some of the ethical dimensions of using social media to increase the number of living kidney donors. Social media provides a platform for changing non‐identifiable ‘statistical victims’ into ‘real people’ with whom we can identify and feel empathy: the so‐called ‘identifiable victim effect’, which prompts charitable action. We examine three approaches to promoting kidney donation using social media which could take advantages of the identifiable victim effect: (a) institutionally organized campaigns based on historical cases aimed at promoting non‐directed altruistic donation; (b) personal case‐based campaigns organized by individuals aimed at promoting themselves/or someone with whom they are in a relationship as a recipient of directed donation; (c) institutionally organized personal case‐based campaigns aimed at promoting specific recipients for directed donation. We will highlight the key ethical issues raised by these approaches, and will argue that the third option, despite raising ethical concerns, is preferable to the other two.

## 1 BACKGROUND

Patients on the kidney transplant waiting lists face an uncertain future, with long waiting times and extended periods of dialysis treatment being typical experiences. Kidney transplantation is perceived to offer significant benefit, in terms of both long‐term survival^1^ and quality of life^2^ for recipients and, because of its cost‐effectiveness in comparison to dialysis,^3^ to the welfare systems more generally. Many of those listed for transplants will never be offered a kidney, and worldwide, thousands of people die each year waiting for a kidney transplant. New ways to increase the number of successful kidney transplants are constantly being explored. One such way is to increase the number of donors, which can be achieved by increasing the number of deceased donors, or increasing the number of living donors.

The former is limited by the manner in which people die, what precipitated death and whether consent is obtained. Patients have to die in relatively controlled circumstances for donation to proceed,^4^ and some conditions, such as cancer, may be regarded as contraindications for donation.^5^ Regardless of donation consent rates, relatively few people die in circumstances compatible with donation. Medical advances have made it possible to expand the eligibility criteria for donation, to include older patients or those with certain morbidities that would previously have precluded donation. The so‐called ‘marginal’ organs from expanded criteria donors tend to produce worse outcomes for patients than non‐marginal organs,^6^ so although more patients receive transplants, increased reliance on marginal organs is an imperfect solution to the organ shortage.

The number of living kidney donors is less constrained by *ability* to donate—the proportion of healthy adults who could potentially donate a kidney is greater than the number of those who die in circumstances compatible with donation. Instead, the number of people *willing* to donate is the limiting factor. This is unsurprising—a healthy person may be reluctant to undergo surgery that is of no clinical benefit to them, and the majority of living donors, in most countries, donate to their family or close friends. The immediate risk of mortality associated with living kidney donation (LKD) is very low (one study suggests 3.1 deaths per 10,000 donors in the 90 days following donation^7^), but it is nonetheless a greater risk than not donating. There are also risks of morbidities (including a greater risk of end‐stage renal disease^8^), and there are the burdens of donation (potentially loss of income, discomfort, and so forth) to consider. Moreover, people may not be aware of the need for more kidney donors, or that they could be a living donor, or they may regard the shortfall in kidneys as somebody else's problem.

Kidneys from living donors tend to produce better transplant outcomes than kidneys from deceased donors,^9^ and the large pool of potential donors means that the benefits of promoting living donation would be significant for recipients. In the United Kingdom, National Health Service Blood and Transplant (NHSBT) is responsible for policy in relation to transplantation. NHSBT has published a strategy document for increasing LKD, outlining several goals and outcomes and how these will be achieved.^10^ Its key aims are as follows:

1. To increase living donation kidney transplantation (LDKT) activity for both adult and paediatric recipients, ensuring that donor safety and welfare is consistently sustained through best clinical practice.
2. To maximize patient benefit by ensuring that all suitable recipients have equity of access to LDKT and that the principle of ‘transplant first’ is embedded in best clinical practice across the United Kingdom.
3. To maximize the opportunities for suitable donors and recipients to contribute to and benefit from the shared living donor pool by ensuring that the National Living Donor Kidney Sharing Schemes are both clinically and cost‐effective.

As the immediate risks associated with LKD have decreased, the use of living donors has apparently become more acceptable, particularly where kidneys are donated to close family members.^11^ ‘Altruistic’ LKD, where the donor is not related to the recipient, nor a friend of long‐standing, remains controversial,^12^ with concerns raised about the motivations of people willing to undergo surgery in order to help strangers, the extent to which donation could be in a healthy person's best interests, and correspondingly whether participation in living donation contravened the primary duty of doctors to ‘do no harm’. The number of altruistic donations is generally increasing, and the fact that altruistic LKD no longer requires additional psychiatric assessment,^13^ and is mentioned in NHSBT strategy, suggests that it is becoming less controversial.

Exposing healthy people to *any* risk seems non‐ideal when there is an adequate alternative that exposes them to no risk. So although in this article we will explore how social media could be used to increase rates of LKD, we do so on the assumption that significant efforts should also be made to increase deceased donation with the ultimate goal being to arrive at a situation where LKD is unnecessary. The NHSBT strategy presupposes that it is currently desirable to increase rates of LKD, so in this article we explore the role of social media in achieving this aim.

## 2 MEDIA CAMPAIGNS AND SOLICITING EMPATHY

Given the potential risks and burdens, becoming a living donor requires a greater degree of motivation than registering a desire to be a donor after one's death. The promotion of living donation to help a stranger may therefore require more than simply raising awareness; and may also need to provide an individual with sufficient motivation to volunteer. Joining a deceased organ donor register (ODR) commits one to being a ‘hypothetical’ organ donor; most people who join will never go on to donate organs because so few will die in circumstances compatible with donation. When one volunteers for LKD, there is a good chance that the offer will be taken up. To be effective, therefore, the promotion of living donation must not just generate support for a worthy cause, but ensure that this support is followed up with a donation.

Throughout this article we will draw a distinction between two broad approaches to promoting LKD. First, the ‘awareness‐raising’ approach, based on the provision of generalized information and statistics regarding the need for, and benefits of, organ donation. Second, the ‘personalized’ approach, where there is a much greater emphasis on a specific patient's need for a transplant and a potential donor's ability to help that specific person. Awareness‐raising has long been the approach used to promote deceased donation. This has generally taken the form of campaigns, where, via various channels, the public are told about the number of people waiting for transplants and the benefits that organ donation can provide. Awareness‐raising via delivery of statistics and education has not resulted in sufficient kidney donations for everyone who needs one. A more personalized approach has also been used, but has proven controversial. The 2008 ‘Kill Jill’^14^ campaign in Scotland was controversial in part because it traded on emotion, using an apparently identifiable individual and giving the audience 20 s to decide whether or not to save her. Such advertising efforts have generally focussed on increasing rates of deceased donation, but NHSBT's new strategy to promote living donation signifies a departure from this.

Some organ donation campaigns have featured individual ‘case studies’ of people waiting for transplants, explaining their situations and highlighting the benefits that a transplant could provide—sometimes called ‘stories of hope’.^15^ The use of case studies is presumably intended to appeal to people by making the human impact of the organ shortage clearer, with the aim of generating empathy in order to motivate people to take action to help. ‘Empathy’ can describe many complex phenomena, but is most commonly used to refer to ‘affective empathy’, defined as ‘feeling the way another feels, or having congruent emotion, *because* the other feels that way’.^16^ This form of empathy includes the perspective‐taking common to other forms (e.g., cognitive empathy), but is distinct by virtue of the emotional reaction to the assumed mental state of another. The empathy–altruism hypothesis widely described and evidenced in psychological literature suggests that empathic concern for another can lead to altruistic motivation to further that other's welfare.^17^ Although the precise nature of the altruism that motivates organ donation is debated,^18^ it is reasonable to assume that the motivation behind most living donation is at least partly other‐regarding, so if empathy can result in other‐regarding behaviour, one can reasonably speculate that increasing the empathy felt for potential kidney recipients could lead to an increased number of kidney donations.

Moreover, although the concept remains disputed, some psychological research suggests the existence of an ‘identifiable victim effect’,^19^ where people act differently towards identifiable victims than to non‐identified ‘statistical victims’. Considering the perspectives of others is a necessary precondition for empathic emotional responses to situations,^20^ and it is easier to consider or assume the perspective of another person when they are an identified individual, rather than a mere statistic. It has been claimed that ‘[i]dentifiable victims seem to produce a greater empathic response, accompanied by greater willingness to make personal sacrifices to provide aid’.^21^ One example of this effect is the response to photos of the drowned Syrian refugee child, Aylan Kurdi, which ‘went viral’ on social media.^22^ Although the refugee crisis had been ongoing for some time prior to Aylan's death, and it was widely known that many people, including children, were dying while trying to flee to safety, the widespread publication of photos with an identifiable victim resulted in a notable (short term, at least) change in attitudes from the public, the press, and politicians. One can appeal to people by providing facts, figures, and impartial generalized reports, but something that prompts a stronger and immediate emotional reaction may be more effective at motivating them to provide a solution. There is a scarcity of empirical research on this approach within the context of organ donation,^23^ but using ‘identifiable victims’ within a personalized approach to promoting donation may therefore be an effective way to increase LKD.

## 3 IS THE PERSONALIZED APPROACH MANIPULATIVE?

Appealing to emotion rather than directly to people's rational capacities may raise concerns about manipulation, but *allowing* emotion to play a motivating role in donation is nothing new. Motivation for living donation between family members, for instance, is likely to involve a significant emotional component.^24^ The same is also true when decisions are made by families in relation to deceased donation; there is no requirement that these decisions must be made completely rationally, absent emotion. Emotional responses may even sometimes promote rational decision‐making. Feeling distressed because another person feels distressed, for example, could prompt appropriate other‐regarding concern.^25^ Despite this, deliberately provoking an emotional reaction to motivate living donation may be different from allowing emotion to play a role in donation. It is generally accepted that a decision to become a living donor should be informed and made freely without coercion, and this may appear to discount intentional manipulation of emotions.

Any marketing material designed to promote organ donation is attempting to make people act in a certain way, but this is not always ethically problematic. Black and Forsberg suggest that objections on the grounds of manipulation comprise two claims. First, a descriptive claim that someone induces someone else to do something. And second, an evaluative claim that this is done in an unethical way.^26^ So while advertising for organ donors is descriptively manipulative—as highlighted by Black and Forsberg, it would be extremely wasteful to use resources if the aim were not to change behaviour^27^—it is not necessarily manipulative in the evaluative sense. For the purposes of this discussion, we will assume that something is manipulative in the evaluative sense if it significantly undermines autonomy.^28^ The important question, therefore, is whether donation influenced, in part, by emotion can be considered sufficiently autonomous.

Although there are many competing accounts of autonomy, common to all of them is a general sense of self‐governance, so external influence is naturally likely to be in some tension with this. Blumenthal‐Barby presents a schema of influence, including (ordered from the least to the most morally problematic) (a) reason and argument, (b) non‐argumentative influence (reason‐bypassing type), (c) non‐argumentative influence (reason‐countering type), (d) omission, and (e) force or severe threats. The mid‐point, ‘non‐argumentative influence (reason‐countering type)’, is defined as ‘influence that operates by countering a person's reasoning capacities, with examples including social norms/pressures, inducing affective states, playing on desires’.^29^ A single act of influence can fall into multiple categories, but a personalized approach designed to prompt empathic responses could be classified as a reason‐countering type of non‐argumentative influence. Relationships between reasons, emotion and autonomy are widely debated within philosophy, but it has been suggested that non‐argumentative influence compromises some conceptions of autonomy.^30^ Although it seems unlikely that appealing to empathy to promote organ donation is *coercive*, as no obvious restrictions are placed on a potential donor's choices, influencing people in this way could be considered to compromise their autonomy to a lesser extent.

Faden, Beauchamp, and King argue that the extent to which a person's autonomy is compromised depends on how difficult the manipulated person finds it to resist, and how much the non‐rational influence interferes with the manipulated person's substantial understanding of the issue at hand.^31^ In the case of organ donation messages, an appeal to emotions such as empathy can be considered to ‘direct the agent's attention towards a set of options and make them salient’,^32^ rather than directing someone down an entirely manipulated pathway. One remains free to deliberate and reflect upon the information provided, and to decide for oneself which course of action to take without external pressure. Even if one's motivation to initially come forward as a potential donor was prompted solely by a spontaneous and uncontrollable outpouring of empathic emotion, the protracted process of work‐up for donation makes it unlikely that one would get to the point of donating without reflecting on one's decisions. For these reasons, using a personalized approach and prompting empathic responses with the aim of promoting organ donation does not seem unduly manipulative. Similar claims have been defended elsewhere in the context of famine relief.^33^ This is not to say that such personalized appeals could not be manipulative (putting a seriously ill patient and their distressed family in the same room as a potential donor and asking the potential donor to donate might quite rightly be considered manipulative) but we will only be discussing options at the other end of the spectrum.

## 4 SOCIAL MEDIA

The last decade has seen the rapid increase in popularity of social media. Sites such as Facebook, Twitter, and YouTube have provided platforms for individuals to share their lives with friends and strangers alike. The facility to share all facets of one's life has created opportunities for individuals to draw attention to negative as well as positive life events and experiences. Social media campaigns have proved to be an effective way of generating support for causes, by both individuals and organizations. Crowd‐funding, for example, is used extensively by patients in the United States to raise funds to pay for expensive medical treatment not covered by insurance.^34^ Social media has become very effective at generating groundswell of support, and terms such as ‘going viral’ illustrate how quickly support can build. Given this, it is unsurprising that transplant authorities and individual patients make use of social media in an attempt to generate more organ donations.

Various Facebook pages have been dedicated to campaigns in transplantation. Facebook has the facility for registered donors to share their organ donor status with their friends via their profile.^35^ More recently, organ donation has been promoted using the dating app, Tinder.^36^ Organizations, as well as individuals, have seized the opportunity offered by the immense audience.^37^ Their efforts have largely focussed on promoting deceased donation, but social media can be used in many ways to promote living organ donation; some of which raise awareness, and others use more personalized approaches.

Against this background, we will now explore three ways in which social media could be used to the advantage of the identifiable victim effect by using personalized approaches in order to promote LKD. Our discussion is based on two key assumptions, introduced earlier:

1. That invoking empathy can lead to altruistic actions, such as donating a kidney.
2. That people are more likely to act altruistically towards an identifiable victim than a statistical victim.

Approach 1 *Institutionally organized* case study‐based campaign to promote *non‐directed* altruistic donation.

The use of stories of identifiable patients who have been helped by (or who died needing) a donated organ are used to promote donation in many countries.^38^ By outlining the experiences of people before and after transplantation, the need for and benefits of organ donation are highlighted in a way that relates to a specific individual and encourages empathy. Although they provide an identifiable victim, the resulting donations are not for the person in the adverts (as, in most cases, they have already received a transplant or died). Instead, the patient is featured as an illustration of the good that transplantation can achieve, or the harm that it can prevent. The donated organs are allocated according to the national allocation models, balancing criteria such as urgency, benefit, and waiting time.

This approach to promoting organ donation raises relatively few ethical issues in its current form, and the same would be true if this was transferred over to social media. Nonetheless, transplantation is ‘marketed’ with a particularly positive spin. The types of people featured in these case studies are ethnically diverse, presented as having positive attributes (active, hardworking good parents) and illnesses unrelated to lifestyle. Understood as objective awareness, raising this could be regarded as deceptive, as it attempts to influence behaviour by providing potentially misleading information: If someone chooses to be a donor on the understanding that their donation is going to help someone similar to the patients featured in the marketing materials, they may be concerned to learn that their donation has been allocated to someone who they would not be so keen to help. However, understood as a mechanism for evoking general empathy for the plight of those in need of organs, it seems less deceptive, since most people waiting for transplants will be experiencing relatively similar plights. But then as a personalized approach taking advantage of the identifiable victim effect, this approach falls someway short. Although the patients featured in marketing materials are identifiable and may invoke a sense of empathy, donors are still being asked to help non‐identified victims, so it seems likely that some of the identifiable victim effect may be lost.

Approach 2 Case study‐based campaigns *organized by individuals* to promote *directed* altruistic donation.

This approach is becoming popular on Facebook and dedicated websites such as matchingdonors.com.^39^ Pages and profiles can be set up (often by the patients themselves, but sometimes by their family or friends), which tell a patient's story with the aim of prompting a donation specifically to that individual. Such pages often feature detailed descriptions of the patient, their illness, and their life history, and the more successful pages are presented in a way that tugs at the viewer's heartstrings with the aim of eliciting empathy. Key aspects of their lives are brought to the fore in an apparent attempt to resonate with ‘browsers’ with shared values or interests. For instance, many pages display photos of adult patients surrounded by their children and grandchildren, with accompanying text highlighting how important they are to each other—something that people with close family members can relate to. The success of this general approach is evidenced by a steady stream of directed altruistic donors in the United States, where it has been permitted for a long time. The approach is newer in Europe, but has resulted in donations in the United Kingdom and the Netherlands. Several European countries, including France, Germany, and Greece, prohibit non‐directed altruistic donation, and these same countries do not currently permit directed altruistic donation either.^40^

This form of directed living donation (also known as publically solicited donation [PSD]) has raised concerns. The (largely) European network Ethical, Legal, and Psychological Aspects of Transplantation's (ELPAT) position paper highlights potential for unfair allocation, damaged public perceptions, and links to the organ trade.^41^ Similar concerns have been raised by others,^42^ but such solicitation is legal in the United Kingdom (provided there is no payment involved), and the UK's Human Tissue Authority has recently included guidance on their own website for potential donors and recipients thinking of using social media and dedicated websites.^43^ The potential negative dimensions of this form of donation have to be balanced against potential benefits to recipients of increasing the numbers of successful transplants, which could be significant if this approach was promoted more widely.

For patients in need of a transplant, PSD is an opportunity to actively improve their own situation, something they are arguably entitled to do, given the costs and burdens of not gaining an organ.^44^ Those who do not have a suitable living‐related donor are likely to spend a long time on waiting lists, and in many cases also on dialysis. Actively marketing and campaigning for oneself as a desirable target for potential donors provides two potential benefits: one may obtain the benefits of a transplant, and one also regains some sense of control over one's own destiny. This latter benefit may accrue regardless of the outcome. Alternatively, however, it could result in a heightened sense of abandonment, especially on dedicated sites where other ‘advertisers’ attract donors but one's own efforts are unsuccessful. This is, after all, a space where people are actively and openly competing for a scarce resource and where efforts to help oneself (and not others) are rewarded. PSD creates something of a ‘beauty contest’,^45^ where patients put themselves in the shop window and potential donors are not just free to choose whether to donate or not, but also able to browse and choose a recipient on whatever grounds they like. This may further the preferences of some donors, but also brings concerns about justice into play. Although everyone waiting for a kidney may be free to advertise, some patients are better placed and able to promote themselves more effectively than others. This poses a greater ethical challenge in some health systems than in others. For instance, in welfare systems where transplant services and the allocation of organs are run by the state, PSD may detract from the number of kidneys available to the general allocation pool. PSD disrupts the usual criteria that balance clinical need, benefit, and waiting time. Instead kidneys may be ‘allocated’ according to distinctly non‐medical factors, such as religion, social value, and how photogenic the patients are. There are related issues stemming from unequal access to social media, varying social media savviness, and ability or otherwise to fund impressive and engaging campaigns.

Two claims against this form of LKD can be posited, then:

1. It is wrong for people to choose recipients on the basis of non‐medical, or otherwise irrelevant criteria.
2. Allocation according to donors’ personal criteria will result in an unjust allocation of organs, skewed in favour of those who, often by luck alone, appeal to donors or who are able to market themselves most effectively.

In response to claim 1, much has been written on directed donation within the context of deceased donation,^46^ and many of these arguments can also be applied to living donation. One can grant that it is generally preferable for organs to be allocated according to criteria designed to promote fair access, but this does not in itself provide a reason to prevent donations that can *only* be allocated according to other criteria. Faced with the choice of allocating a kidney according to potentially arbitrary criteria, or not having the kidney available for transplantation at all, it is arguably wasteful to take the latter option.^47^ Living‐related donation is also widely accepted, so directed donation is not considered objectionable per se.

In response to claim 2, it is not yet clear what the impact of PSD will be in countries which have centralized allocation systems. Given existing evidence about the public's preferences for organ allocation, where factors such as social value, moral deservingness, and past behaviours have been found to feature,^48^ it is reasonable to assume that encouraging a ‘free market’ in charitable giving on social media has the potential to result in significant inequalities of the kind that central allocations systems are designed to prevent. Promoting and encouraging PSD would therefore represent a risky strategy. Its potential to evoke empathy may result in more living donations, but there is a significant risk that this will come at a cost to the fairness of the allocation system, which, in turn, may undermine trust in the transplantation system. It is perhaps telling that countries that do permit PSD appear to tolerate rather than promote it.

Approach 3 *Institutionally organized* case study‐based campaigns to promote *directed* altruistic donation.

We have thus far explored two ways in which social media can be used to elicit empathy and take advantage of the identifiable victim effect to promote kidney donation. We will now consider a third approach that is perhaps controversial but offers clear advantages over the other two approaches. As far as we are aware, this approach is not currently used in any country.

We have argued that although PSD could result in additional donors, promoting it via Approach 2 would involve a significant risk. Approach 2 empowers donors and recipients, but in doing so it relinquishes control over important aspects of organ donation and allocation. Alternatively, transplant authorities could harness social media to make direct appeals on behalf, and with the consent, of specific wait‐listed individuals selected according to similar principles used to allocate organs in their jurisdiction, and bringing to bear for each selected individual all of their sophisticated marketing resources. In doing so, the benefits of social media are obtained without relinquishing control over the aspects of donation and allocation that would make promoting Approach 2 such a risk.

For instance, transplant authorities could promote only the stories of urgent patients at the top of waiting lists, thereby increasing their likelihood of receiving a transplant before they die or become too ill to benefit from a transplant. Alternatively, they could promote those who have waited, or are predicted to be waiting, a long time for a transplant, such as patients of Black or Asian ethnic origin.^49^ A further option could be to promote the cases of those involved in altruistic chains of donation. Altruistic chains occur when Donor A is willing to donate to Recipient A, but is instead only a match for Recipient B, who also has a willing donor, Donor B, who is only a match for Recipient C. Finding Donor X online, who is a match for Recipient A, can start a chain of donations (Donor X to Recipient A, Donor A to Recipient B, Donor B to Recipient C, and so on), which means that several otherwise incompatible donor/recipient pairs are able to receive and donate. These chains often require a single donation to be ‘fed’ into the chain to create a domino effect so that multiple patients receive transplants. The decision on precisely what to prioritize is not central to our argument. The salient point is that effort could be directed impartially and fairly by harnessing the power of empathy evoked online to motivate donation. Although potential donors may feel empathy towards only certain individuals and choose recipients according to criteria that are potentially morally irrelevant, by ensuring that potential donors are exposed to patients who meet the morally relevant criteria, the justice of the allocation system need not be compromised.

This approach also allows financial resources to be used more effectively. A significant problem with PSD is that it is sometimes used to ‘find the needle in the haystack’ by patients who, for various medical reasons (such as rare blood type, or Human leukocyte antigen [HLA] sensitization),^50^ are particularly unlikely to find a suitable matching donor. A single patient may attract tens of potential donors, each of which would have to be tested for compatibility, even though the likelihood would be that none of them was a suitable match. The resource implications are significant, so transplant authorities could choose to promote only the stories of patients who stand a good chance of finding a match, thereby swiftly removing them from the waiting list. Nevertheless, there would remain a chance that the system we propose might motivate many people to come forward as potential donors only to find that they are not compatible with their preferred recipient. These people may be lost to the donation system if they cannot help their chosen recipient (and this would be an argument against this approach if these were donors who, without PSD, may have been willing to donate to a non‐specified recipient). This would be undesirable given that they have already shown willingness to debate and used resources involved in tissue‐typing. One solution to this would be to develop a database, akin to that operated by Anthony Nolan in the United Kingdom,^51^ where potential donor details and tissue‐typing results are stored. With appropriate consent, potential donors could remain on this database and be notified when stories of patients with whom they are a match are promoted. The potential donor would then be free to choose whether they wanted to donate a kidney to this patient. Detailed consideration of a living donor register is beyond the scope of this article, so we raise it merely as one possible means of keeping initially incompatible donors engaged with the possibility of donating in the future.

By taking back control over advertising for LKD, transplant authorities would also be able to have greater control over the content of advertisements for donors. Whereas there have been concerns about the beauty contest created by PSD, an institutionally organized system of LKD could ensure that only certain factors featured in the descriptions of patients. Careful consideration would have to be given to precisely what things should be included in descriptions of patients, as it will be necessary to balance privacy (by excluding extraneous personal factors) with the effectiveness with which the promotion of the individual engages with the public and evokes empathy. Additionally, by controlling the content of appeals, the authorities would be ensuring that the content is accurate and not misleading to potential donors. This, in turn, would limit the potential for exploitation on both sides, and help to maintain public trust in the system. There is a possibility, however, that institutionally organized campaigns could lose the ‘individuality’ that appeals to some donors, or that too much ‘official’ institutional input may deter some donors who want to donate outside of conventional systems, so these considerations would have to be balanced carefully.

A further advantage of this approach is that it would be easier to monitor communication between potential donors and recipients. There have been concerns that the use of social media can be manipulated to permit trade in organs, which is prohibited under the Declaration of Istanbul.^52^ We have also suggested that they leave scope for deception—as no one vets the veracity of the information provided by recipients. At present, donors and recipients identified through social media are able to contact each other through channels of their choice, and it is extremely difficult to monitor and police this communication. Although websites such as matchingdonors.com report to relevant authorities any discussions that mention payment, there is nothing to stop users of the site exchanging other contact details and starting conversations about payment away from the main website. While it is impossible to completely safeguard against payment, an institutionally organized system could limit the sharing of contact details, and encourage communication solely through its own channels.

## 5 OBJECTIONS

We can foresee several objections to Approach 3. First, by engaging empathy to promote LKD, transplant authorities are arguably encouraging donation motivated by potentially irrelevant or morally suspect criteria such as the recipients’ race, religious beliefs, or social status. Motivation behind donation has been discussed at length in relation to directed deceased donation, and it has been repeatedly argued that too much emphasis is placed on impartial altruism as being the sole acceptable motivator, and that, in any case, it is very difficult to be certain of donors’ motivations.^53^ Moreover, one undesirable effect of suspect motivation—the disruption of fair allocation—is mitigated by the use of fair criteria to decide which cases are promoted. Thus, organs go where they ‘should’ even if those giving them are indifferent, or even hostile, to impartial allocation.

Second, there is still a risk that some sorts of people will benefit more than others. As with PSD described in Approach 2, some patients may be less likely to attract willing living donors than others. There may, however, be significant direct benefit for all patients. Although a specific campaign might not prove fruitful for those who are less ‘beautiful’ in the sense implied by Neidich et al. and Moorlock, if Approach 3 results in additional donations overall, it will remove patients from waiting lists. Having fewer patients on waiting lists improves the situation for everyone remaining on the waiting list. Those featured in the campaigns would still have access to organs’ normal channels. Therefore, although Approach 3 may not benefit everybody equally, it may provide some benefit to everyone.^54^ It supports a more just allocation system than PSD under Approach 2, which is already permitted.

Third, one might think it distasteful to have patients openly and literally competing with one another for resources. A Dutch television programme caused controversy in 2007 when it showcased several potential organ recipients and allowed viewers to vote on which patient they felt should receive a transplant.^55^ The programme transpired to be a hoax, produced with the intention of raising awareness of the organ shortage, but it highlights the potential for negative public reactions. The reality, however, is that potential recipients are *already* competing with each other, whether actively or passively.^56^ Likewise, donors already choose who receives transplants (in living‐related donation, and PSD), and therefore to some extent, are already choosing who lives and who dies. It may be that state involvement in active competition is considered objectionable, but the state is already *involved* as it ultimately facilitates and reaps the benefits of the donations and transplants that currently arise from it. Moreover, our proposed system could side‐step some of the less appealing aspects of direct competition. For instance, promoting only a single patient at a time (for a limited period of time, say a week) would mean that potential donors would not be choosing between several competing identifiable recipients. If it was still deemed advantageous to promote several patients at the same time, careful selection could ensure that direct competition was minimized, for instance, by ensuring patients had different tissue‐types.

A further issue is how Approach 3 could operate in conjunction with Approach 2. It would be possible (albeit difficult) to only permit Approach 3 and prohibit individuals from launching their own ‘independent’ campaigns. This would, however, limit the scope for patients to help themselves out of their own dire situations, and although this could perhaps be justified, it may be viewed as a backward step in countries that already permit Approach 2. It may also prove practically difficult to monitor and prevent such campaigns, given the large and often uncontrollable nature of the internet. It would be possible to refuse to proceed with transplants arising from donors and recipients matched via Approach 2,^57^ but turning away willing donors could be considered a waste of potential benefit. Another option would be to make patients’ access to Approach 3 conditional on them not attempting to solicit donors through independent channels.^58^ If Approach 3 could be demonstrated to be an effective way of recipients finding donors, such a condition may result in Approach 2 becoming less popular. Some people may still choose independent channels over Approach 3, but if the institutionally organized campaign system was marketed as the ideal and ‘official’ portal for finding potential organ recipients, Approach 2 may also become less appealing to potential donors. Again, we need not endorse a particular position here, other than to show that there are different possibilities. Determining which option is preferable requires empirical evidence, which is currently lacking.

## 6 CONCLUSIONS

We have discussed three approaches to using social media to promote organ donation. Approach 1 raises the fewest ethical issues, but is likely to be least effective. Approach 2 is ethically problematic in some respects, but is currently permitted in some countries. Despite it being permitted, it is generally not actively promoted, possibly due to the underlying ethical concerns, and the risks involved in leaving important aspects of donation/allocation in the hands of individuals rather than impartial organizations. Approach 3 is novel, and admittedly controversial. It does, however, offer clear advantages over Approach 2 in terms of preserving a relatively just allocation system and keeping various aspects of the process under the control of transplant authorities. Further research exploring responses to social media organ donation campaigns, as well as motivations behind PSD, more generally would be extremely helpful for establishing how our suggested approach could be best used. We accept that establishing such a system could be resource‐intensive, and that ongoing costs may also be high, but these costs should be considered against the ongoing cost of maintaining patients waiting for transplants. The precise implementation of Approach 3 would need careful consideration, but the important factor is that these details remain under the control of transplant authorities. Approach 3 appeals to empathy to do the motivating, but does so in a way that minimizes disruption to desirable allocation policies. Approach 3, if implemented correctly, could increase rates of LKD without significantly undermining the justice of kidney allocation.

## CONFLICT OF INTEREST

The authors declare no conflict of interest.

## Footnotes

1
Oniscu, G. C., Brown, H., & Forsythe, J. L. (2005). Impact of cadaveric renal transplantation on survival in patients listed for transplantation. *Journal of the American Society of Nephrology*, *16*(6), 1859–1865. 10.1681/ASN.200412109215857921

2
Liem, Y. S., Bosch, J. L., & Myriam Hunink, M. (2008). Preference‐based quality of life of patients on renal replacement therapy: A systematic review and meta‐analysis. *Value in Health*, *11*(4), 733–741, Wyld, M., Morton, R. L., Hayen, A., Howard, K., & Webster, A. C. (2012). A systematic review and meta‐analysis of utility‐based quality of life in chronic kidney disease treatments. *PLoS Medicine*, *9*(9), e1001307. 10.1371/journal.pmed.1001307PMC343939222984353

3
Barnieh, L., Gill, J. S., Klarenbach, S., & Manns, B. J. (2013). The cost‐effectiveness of using payment to increase living donor kidneys for transplantation. *Clinical Journal of the American Society of Nephrology*, *8*(12), 2165–2173. 10.2215/CJN.03350313PMC384839524158797

4
Barber, K., Falvey, S., Hamilton, C., Collett, D., & Rudge, C. (2006). Potential for organ donation in the united kingdom: Audit of intensive care records. *BMJ*, *332*(7550), 1124–1127. 10.1136/bmj.38804.658183.55PMC145955716641118

5
Israni, A., Zaun, D., Bolch, C., Rosendale, J., Snyder, J., & Kasiske, B. (2016). Deceased organ donation. *American Journal of Transplantation*, *16*(S2), 195–215. 10.1111/ajt.1367326755269

6
Saidi, R., Elias, N., Kawai, T., Hertl, M., Farrell, M. L., Goes, N., *…* Ko, D. S. (2007). Outcome of kidney transplantation using expanded criteria donors and donation after cardiac death kidneys: Realities and costs. *American Journal of Transplantation*, *7*(12), 2769–2774. Aubert, O., Kamar, N., Vernerey, D., Viglietti, D., Martinez, F., Duong‐Van‐Huyen, J.‐P., … Loupy, A. (2015). Long term outcomes of transplantation using kidneys from expanded criteria donors: Prospective, population based cohort study. *BMJ*, *351*, h3557. 10.1111/j.1600-6143.2007.01993.x17927805

7
Segev, D. L., Muzaale, A. D., Caffo, B. S., Mehta, S. H., Singer, A. L., Taranto, S. E., … Montgomery, R. A. (2010). Perioperative mortality and long‐term survival following live kidney donation. *JAMA*, *303*(10), 959–966. 10.1001/jama.2010.23720215610

8
Muzaale, A. D., Massie, A. B., Wang, M.‐C., Montgomery, R. A., McBride, M. A., Wainright, J. L., & Segev, D. L. (2014). Risk of end‐stage renal disease following live kidney donation. *JAMA*, *311*(6), 579–586. 10.1001/jama.2013.285141PMC441195624519297

9
Matas, A., Smith, J., Skeans, M., Thompson, B., Gustafson, S., Stewart, D., … Kasiske, B. L. (2015). Optn/srtr 2013 annual data report: Kidney. *American Journal of Transplantation*, *15*(S2), 1–34. 10.1111/ajt.1319525626344

10
NHS Blood and Transplant. (2014). *Living donor kidney transplantation 2020: A UK strategy* Retrieved from http://www.nhsbt.nhs.uk/download/board_papers/may14/Living-Kidney-Donor-transplantation-2020-A-UK-Strategy.pdf

11
Live Organ Donor Consensus Group. (2000). Consensus statement on the live organ donor. *JAMA*, *284*, 2919–2926. 10.1001/jama.284.22.291911187711

12
Truog, R. D. (2005). The ethics of organ donation by living donors. *New England Journal of Medicine*, *353*(5), 444–446. 10.1056/NEJMp05815516079366

13
Human Tissue Authority. (2015). *Guidance to transplant teams and independent assessors* Retrieved from https://www.hta.gov.uk/sites/default/files/Guidance_to_Transplant_Teams_and_Independent_Assessors%20(1)_0.pdf

14
Scottish Government. (2008). *Kill jill ‐ Organ donation* Retrieved from https://www.youtube.com/watch?v=raMabDzN12s

15
Organ Donation Scotland. (2016). *Stories of hope* Retrieved from https://www.organdonationscotland.org/discuss-share/stories-of-hope. Donate Life ‐ North Carolina. (2016). *Stories of hope* Retrieved from https://www.donatelifenc.org/stories-hope. United Network for Organ Sharing. (2016). *Stories of hope* Retrieved from https://www.unos.org/transplantation/stories-of-hope.

16
Kauppinen, A. (2014). *Empathy, emotion regulation and moral judgment* In H. L. Maibom (Ed.), *Empathy and morality*. Oxford: Oxford University Press.

17
Batson, C. D. (2011). *Altruism in humans* New York: Oxford University Press. Toi, M., & Batson, C. D. (1982). More evidence that empathy is a source of altruistic motivation. *Journal of Personality and Social Psychology*, *43*(2), 281. Feigin, S., Owens, G., & Goodyear‐Smith, F. (2014). Theories of human altruism: A systematic review. *Annals of Neuroscience and Psychology*, *1*(1), 1–19.

18
Moorlock, G., Ives, J., & Draper, H. (2014). Altruism in organ donation: An unnecessary requirement? *Journal of Medical Ethics*, *40*(2), 134–138, Saunders, B. (2012). Altruism or solidarity? The motives for organ donation and two proposals. *Bioethics*, *26*(7), 376–381, Wilkinson, T. (2007). Racist organ donors and saving lives. *Bioethics*, *21*(2), 63–74. 10.1136/medethics-2012-100528PMC391321123538329

19
Small, D. A., & Loewenstein, G. (2003). Helping a victim or helping the victim: Altruism and identifiability. *Journal of Risk and Uncertainty*, *26*(1), 5–16.

20
Kogut, T., & Ritov, I. (2005). The “identified victim” effect: An identified group, or just a single individual? *Journal of Behavioral Decision Making*, *18*(3), 157–167.

21
Jenni, K., & Loewenstein, G. (1997). Explaining the identifiable victim effect. *Journal of Risk and Uncertainty*, *14*(3), 235–257.

22
Smith, H. (2015). *The guardian ‐ Shocking images of drowned Syrian boy show tragic plight of refugees* Retrieved from http://www.theguardian.com/world/2015/sep/02/shocking-image-of-drowned-syrian-boy-shows-tragic-plight-of-refugees.

23
Some research has found that providing anonymized demographic characteristics of potential organ recipients increased likelihood to register as a deceased donor (see: Singh, M., Katz, R. C., Beauchamp, K., & Hannon, R. (2002). Effects of anonymous information about potential organ transplant recipients on attitudes toward organ transplantation and the willingness to donate organs. *Journal of Behavioral Medicine*, *25*(5), 469–476), but we are unaware of research relating to living donation. 10.1023/a:102047100783712442561

24
Crouch, R. A., & Elliott, C. (1999). Moral agency and the family: The case of living related organ transplantation. *Cambridge Quarterly of Healthcare Ethics*, *8*(3), 275–287. 10.1017/s096318019980303x10388928

25
We thank an anonymous reviewer for this helpful point.

26
Black, I., & Forsberg, L. (2014). Would it be ethical to use motivational interviewing to increase family consent to deceased solid organ donation? *Journal of Medical Ethics*, *40*, 63–38. 10.1136/medethics-2013-10145124126202

27
Ibid.

28
Blumenthal‐Barby, J. (2012). Between reason and coercion: Ethically permissible influence in health care and health policy contexts. *Kennedy Institute of Ethics Journal*, *22*(4), 345–366. 23420941

29
Ibid: 349.

30
Dworkin, G. (1988). *The theory and practice of autonomy* Cambridge: Cambridge University Press. Noggle, R. (1996). Manipulative actions: A conceptual and moral analysis. *American Philosophical Quarterly*, *33*(1), 43–55.

31
Faden, R. R., Beauchamp, T. L., & King, N. M. (1986). A history and theory of informed consent. Oxford: Oxford University Press.

32
Tappolet, C. (2006). Autonomy and the emotions. *European Journal of Analytic Philosophy*, *2*(2), 45–59.

33
Noggle, *op. cit*. note 30.

34
Sisler, J. (2012). Crowdfunding for medical expenses. *Canadian Medical Association Journal*, *184*(2), E123–E124. 10.1503/cmaj.109-4084PMC327352822231688

35
Facebook. (2016). *Share your organ donor status* Retrieved from https://www.facebook.com/help/416967021677693/

36
BBC News. (2015). *NHS hooks up with dating app Tinder on organ donations* Retrieved from http://www.bbc.co.uk/news/technology-35070858

37
Transplant Australia. (2016). *Transplant Australia Facebook page* Retrieved from https://www.facebook.com/transplantaustralia/

38
NHS Blood and Transplant. (2016). *People who are waiting for a transplant* Retrieved from https://www.organdonation.nhs.uk/real-life-stories/people-who-are-waiting-for-a-transplant/

39
MatchingDonors. (2016). *MatchingDonors home page* Retrieved from http://matchingdonors.com/life/index.cfm

40
Lopp, L. (2013). Regulations regarding living organ donation in Europe. Berlin: Springer.

41
Frunza, M., Van Assche, K., Lennerling, A., Sterckx, S., Citterio, F., Mamode, N., … Dor, F. J. (2015). Dealing with public solicitation of organs from living donors—An elpat view. *Transplantation*, *99*(10), 2210–2214. 10.1097/TP.000000000000066925769072

42
Neidich, E., Neidich, A., Cooper, J., & Bramstedt, K. A. (2012). The ethical complexities of online organ solicitation via donor–patient websites: Avoiding the “beauty contest”. *American Journal of Transplantation*, *12*(1), 43–47, Moorlock, G. (2015). Directed altruistic living donation: What is wrong with the beauty contest? *Journal of Medical Ethics*, *41*(11), 875–879. 10.1111/j.1600-6143.2011.03765.x21951635

43
Human Tissue Authority. (2017). *Matching websites and social media* Retrieved from https://www.hta.gov.uk/guidance-public/living-organ-donation/matching-websites-and-social-media

44
Coupled with the fact that they may be attracting completely new donors who are only prepared to donate to them, so nobody else is deprived of a transplant that they might otherwise have got.

45
Neidich et al., *op. cit* note 42; Moorlock, *op. cit*. note 42.

46
Moorlock, G., Ives, J., Bramhall, S., & Draper, H. (2015). Should we reject donated organs on moral grounds or permit allocation using non‐medical criteria?: A qualitative study. *Bioethics*; Wilkinson. *op. cit*. note 18. 10.1111/bioe.12169PMC497559826132802

47
Moorlock et al., *op. cit*. note 46.

48
Boulware, L., Troll, M., Wang, N. Y., & Powe, N. (2007). Perceived transparency and fairness of the organ allocation system and willingness to donate organs: A national study. *American Journal of Transplantation*, *7*(7), 1778–1787, Sander, S. L., & Miller, B. K. (2005). Public knowledge and attitudes regarding organ and tissue donation: An analysis of the northwest ohio community. *Patient Education and Counseling*, *58*(2), 154–163, Tong, A., Howard, K., Jan, S., Cass, A., Rose, J., Chadban, S., … Craig, J. C. (2010). Community preferences for the allocation of solid organs for transplantation: A systematic review. *Transplantation*, *89*(7), 796–805, Clark, M. D., Leech, D., Gumber, A., Moro, D., Szczepura, A., West, N., & Higgins, R. (2012). Who should be prioritized for renal transplantation?: Analysis of key stakeholder preferences using discrete choice experiments. *BMC Nephrology*, *13*(1), 1, Geddes, C. C., Rodger, R. S. C., Smith, C., & Ganai, A. (2005). Allocation of deceased donor kidneys for transplantation: Opinions of patients with ckd. *American Journal of Kidney Diseases*, *46*(5), 949–956.

49
This is a particular problem in the United Kingdom, but similar issues occur in other countries with a history of immigration: Randhawa, G. (2012). Renal health and transplantation: A focus on ethnicity. *Journal of Renal Care*, *38*(s1), 109–114. 10.1111/j.1755-6686.2012.00277.x22348370

50
Sanfilippo, F. P., Vaughn, W. K., Peters, T. G., Shield, C. F., Adams, P. L., Lorber, M. I., & Williams, G. M. (1992). Factors affecting the waiting time of cadaveric kidney transplant candidates in the united states. *JAMA*, *267*(2), 247–252. 1727521

51
Anthony Nolan. (2016). *Anthony Nolan home page* Retrieved from https://www.anthonynolan.org/

52
Declaration of Istanbul. (2016). *Declaration of Istanbul home page* Retrieved from http://www.declarationofistanbul.org/

53
Moorlock et al., *op. cit* note 18. Saunders, *op. cit*. note 18.

54
This approach may therefore provide a form of Pareto benefit. See, for examples: Cohen, G. A. (1995). The pareto argument for inequality. *Social Philosophy and Policy*, *12*(1), 160–185. Rawls, J. (1971). *A theory of justice* Cambridge, MA: Harvard University Press. Saunders, *op. cit*. note 18.

55
BBC News. (2007). *Outcry over TV kidney competition* Retrieved from http://news.bbc.co.uk/1/hi/entertainment/6699847.stm

56
Moorlock, *op. cit*. note 42.

57
Anecdotally we have heard of donors and recipients matched through certain dedicated websites being prevented from going ahead with donation.

58
We again thank an anonymous reviewer for this suggestion.

